# Sexual Dimorphism in *Sturnira lilium* (Chiroptera, Phyllostomidae): Can Pregnancy and Pup Carrying Be Responsible for Differences in Wing Shape?

**DOI:** 10.1371/journal.pone.0049734

**Published:** 2012-11-14

**Authors:** Nícholas F. de Camargo, Hernani F. M. de Oliveira

**Affiliations:** 1 Programa de Pós-Graduação em Ecologia, Laboratório de Ecologia de Vertebrados, Departamento de Ecologia, Instituto de Ciências Biológicas, Universidade de Brasília, DF, Brazil; 2 Laboratório de Sementes e Viveiros Florestais, Departamento de Engenharia Florestal, Universidade de Brasília, DF, Brazil; University of Western Ontario, Canada

## Abstract

Competition is one of the most cited mechanisms to explain secondary sexual dimorphism in animals. Nonetheless, it has been proposed that sexual dimorphism in bat wings is also a result of adaptive pressures to compensate additional weight caused by fetus or pup carrying during the reproductive period of females. The main objective of this study is to verify the existence of sexual dimorphism in *Sturnira lilium* wings. We employed geometric morphometrics techniques using anatomical landmarks superimposition to obtain size (Centroid Size) and shape variables of wings, which were reduced by Linear Discriminant Analysis (LDA). We also employed classical morphometrics using wing length measurements to compare efficiency between these two morphometric approaches and make comparisons using wing area measurements. LDA indicated significant differences between wing shapes of males and females, with 91% (stepwise classification) and 80% (leave-one-out cross validation) of correct classification. However, the size variable obtained did not contribute to such classifications. We have observed larger areas in female wings, but we found no differences in wing length measurements and no allometric effects in wing length, shape and area measurements. Interestingly, our study has provided evidences of morphological differences where classical morphometrics have failed. LDA and area measurements analyses revealed that females have a different area distribution in distinct portions of the wing, with wider dactylopatagia and plagiopatagia, and wingtips more triangular than males. No differences in body length or relative wing length were observed between the sexes, but pregnant females have more body weight than non-pregnant females and males. Our findings suggest that sexual dimorphism in the wing shape of *S. lilium* is probably related to the increase in flight efficiency of females during reproductive period. It decreases wing loading in specific portions of the wing and reduces energy cost to maintain a faster and maneuverable flight.

## Introduction

Bats are unique among mammals in their capacity for powered flight. Flight is a form of locomotion that enables foraging over large areas and in areas of difficult access, and also allows migration over large distances [1]. Nevertheless, this type of locomotion requires high-energy costs and, therefore, a strong selection must take place on wings as a way to minimize such costs [2]. As such, selection can favor optimal design of wings for locomotion with less effort in accordance with ecological and physiological characteristics of each species [1]–[5].

Size and shape of structures in organisms are features well used to verify differentiation of species [6]–[8], population variations [9]–[11] and make ecological inferences [3], [4], [6], [12]. The employment of techniques that aim at the maximization and the detection of morphological differentiation of bat wings has been shown to be useful as a way to comprehend the dynamics of flight [4], segregation and coexistence in space [13], [14]. To this end, morphometric parameters that take into consideration the mechanics and aerodynamics were proposed to verify the flight performance of bat species that explore habitats differently and have different flight styles [4], [15]–[17]. For example, insectivorous bats of open habitats have long and narrow wings [4], [16], [17]. As these animals pursue agile and flying preys, their wing shape generates a constant and fast flight, but with limited maneuverability. On the other hand, frugivorous and insectivorous bats from complex and closed habitats (i.e. forest environments) require high maneuverability to explore these areas. To such purpose, they usually have short and/or broad wings [4].

Bat wings can be divided in different anatomical features: dactylopatagium, propatagium, plagiopatagium and uropatagium; and each of these parts have different roles in the flight. While dactylopatagium is related to power generation and propulsion of the bat in the air, plagiopatagium is related to the maintenance of this generated force. On the other hand, propatagium and uropatagium are related to the adjustment of flight height, and for some insectivorous bats, uropatagium is used for insect capture during flight [18].

Despite efforts employed to verify morphological differences in bat wings according to ecological characteristics, intraspecific level differences have not been studied in detail. The sex of animals is one of the factors that may influence ecological and morphological characteristics. In this aspect, insectivorous bats were the most studied up until now [19]–[23].

Sexual dimorphism in bats related to body size, skull morphology and, especially, to the forearm length was found in several studies (see ref. [24] for a list of studies). Among the main selective pressures on secondary sexual dimorphism are: competition for resources among females (big mother hypothesis), reduction in competition for resources among males and females, and competition among males for partners (sexual selection) [24]. However, it has previously been proposed that females may present larger body size (including wings), or only larger wings, as a way to compensate the additional weight of carrying a fetus or newborn bat [19], [20], [23]. With the increase of wing area comes a decrease in wing loading (grams per square centimeter of wing area) [1] and therefore, it does not compromise the dynamics of flight during the reproductive period.

Although morphometric parameters related to size have been tested to verify sexual dimorphism in bats [19]–[23], shape analysis, with attention to variation of anatomical landmarks in the wing, was only explored in four species of vespertilionids [25]. Analysis using anatomical landmarks to evaluate the shape of structures can be highly relevant. Area measurements and length of bones, commonly used on morphometry of bats [19], [20], [26], [27], do not fully describe differences in shape and neglect covariation of measurements or specific points in different regions of the wing [25].

In this study we verified possible intersexual differences in the wings of *Sturnira lilium* using geometric morphometrics, wing area measurements and classical morphometrics to test the hypothesis that females have a different wing to compensate the extra weight caused by fetus mass during pregnancy and breastfeeding of newborns. Newborns of species in the Phyllostomidae family can have 25% to 38% of the mother’s body weight [28]. Thus, we expect sexual dimorphism to be related to wing characteristics that can provide reduction in wing loading of females [20]. More specifically, we expect that this lower wing loading in females will be a result of larger wing and/or different wing shapes for a more efficient flight. Additionally, since no sexual dimorphisms were found in *S. lilium* by means of classical morphometrics [9], we expect geometric morphometrics to have greater capability to detect these differences between sexes.

## Methods

### Ethics Statement

All captured bats were handled by experienced investigators. The animals were kept in cotton bags until the beginning of data acquisition (see *Geometric Morphometrics Analysis* in *Methods*) to avoid hypothermia. Once data acquisition was performed (1–2 minutes) all animals were immediately released. All the methods listed in this study related to care and welfare, comply with the guidelines recommended by the American Society of Mammalogists [29] and with the requirements of The Brazilian Institute for the Environment and Natural Resources (IBAMA) (Permit No. 12428-2; IBAMA Registration No. 2058788).

### Study Area

Data collection took place from September 2007 to June 2008 in eight areas of the Brazilian savannah (Cerrado), Distrito Federal, Brazil. The Cerrado represents a large neotropical biome (2×10^6^ km^2^) that includes savannas, grasslands and forests subject to a highly seasonal climate with a well-defined rainy season (October to April, when 90% of the precipitation expected for the year happens) [30].

Eight areas were chosen to capture bats: one located at the ecological station of Águas Emendadas (5° 32' S, 47° 34' W), two at the Cerrado Research Center of Embrapa (Brazilian Agricultural Research Agency) (15° 35' S, 47° 42' W), one at the ecological reserve of IBGE (Brazilian Institute of Geography and Statistics) (15° 56' S, 47° 53' W), one at the private farm Solar da Águia (15° 55' S, 47° 49 ' W), one at the private farm Santa Helena (15° 38' S, 47° 47 ' W), one at a private farm next to PNB (Brasília National Park) (15° 56' S, 47° 53' W) and the last one at PNB (15° 4' S, 47° 57' W). All animals were captured in gallery forests, a type of forest formation of the Cerrado that is along the course of rivers and streams of the Brazilian Central Plateau [31].

### Captures

Bats were captured with nine mist-nets (36 mm mesh) to a total of 1998 net-hours. Mist-nets were opened one hour before the sunset and removed after six consecutive hours of sampling. The bats were weighed with the support of a dynamometer of 100 g (Pesola micro-line 20100; precision: 1 g) and a caliper (Eccofer 150 mm 6″; precision: 1 mm) was used to measure the length of the body. For data acquisition of body weight, bats were first placed in cotton bags for approximately 60 minutes, allowing digestion of food in the gut and eliminating a possible bias in body weight caused by food mass. After this procedure, we weighted the animals inside a pre-weighted bag. To avoid ontogeny effects in our results we only analyzed adult specimens. Animals were considered as adults when metacarpal epiphyseal cartilages were no longer visible. Additionally, we determined pregnant females by palpation.

### Geometric Morphometric Analysis

**Figure 1:**
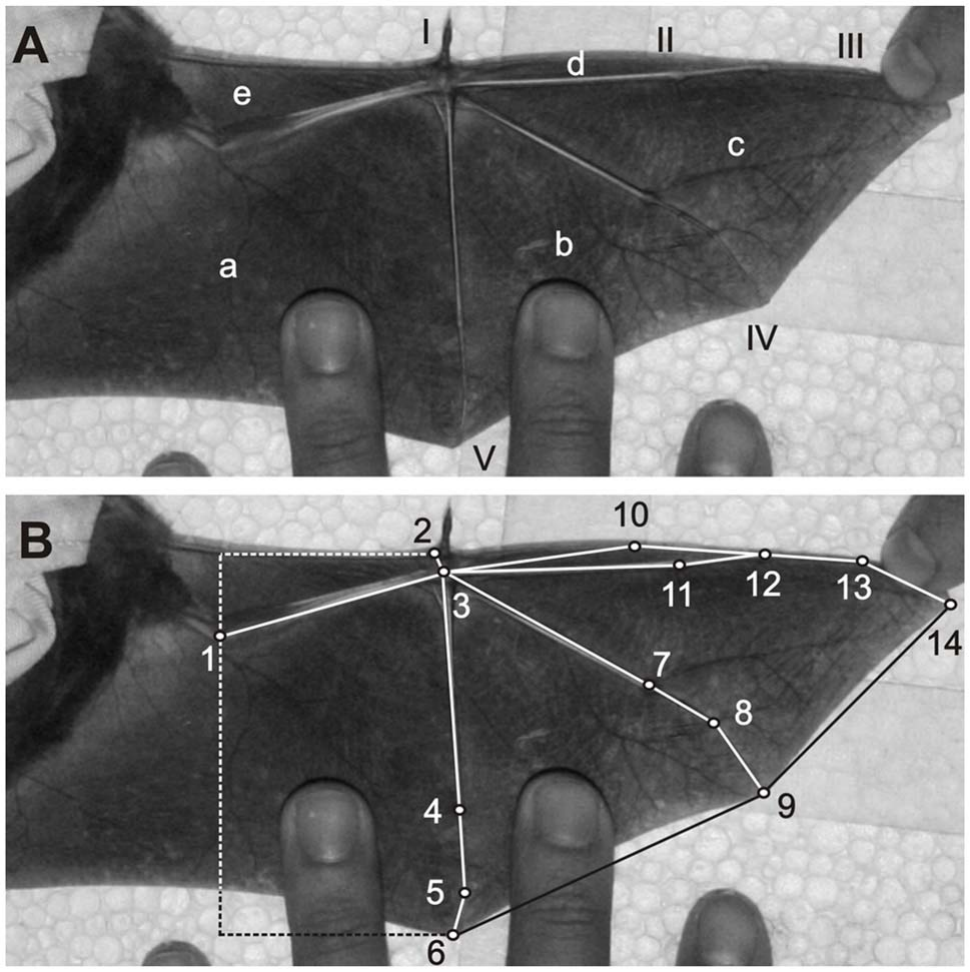
Wing of *Sturnira lilium* revealing the main structures evaluated in this morphometric study. In A) it is possible to observe digits I to V and distinct regions of the wing: (a) propatagium, (b) plagiopatagium, (c) dactylopatagium major, (d) dactylopatagium medius and (e) dactylopatagium minus. In B) it is possible to see the 14 anatomical landmarks used in analyses of partial warps and uniform components of the wing. Anatomical landmarks definition: 1) Articulation between the humerus and radius/ulna; 2) Tissue junction between the propatagium membrane and digit I; 3) Center of the carpus; 4) Articulation between metacarpus and proximal phalange of digit V; 5) Articulation between proximal and distal phalanges of digit V; 6) Tissue junction between distal phalange of digit V and propatagium membrane; 7) Articulation junction between metacarpus and proximal phalange of digit IV; 8) Articulation between proximal and distal phalanges of digit IV; 9) Tissue junction between distal phalange of digit IV and dactylopatagium major membrane; 10) Maximum curvature point of dactylopatagium minus; 11) Articulation between metacarpus and proximal phalange of digit III; 12) Articulation between proximal and intermediate phalanges of digit III; 13) Articulation between intermediate and distal phalanges of digit III; 14) Tissue junction between distal phalange of digit III and dactylopatagium medius membrane.

We performed morphometric analyses from information collected on photos of bat wings. To photograph specimens the left wing of each individual was extended in the center of a styrofoam board covered in cork and photographed with a digital camera (Canon EOS DIGITAL REBEL XT - Canon EF-S 18–55 lens), which was mounted to a tripod. These images were taken with the camera adjusted to the same height and with no zoom effect. For the standardization of wing position, we have considered the fifth finger parallel to the body of the animal and the largest possible stretching of major (digits IV and V) and medius (digits III and IV) dactylopatagium membranes (Figure 1a). Additionally, we considered the maximum angulation between the humerus and the radio/ulna (Figure 1a). The arm extension of bats is related to the stretch capacity of the propatagium membrane. Therefore, if there was any resistance in the extension of the bat’s arm, to avoid injury, we have considered this as the maximum angulation between humerus and radio/ulna. Wing images were taken of non-pregnant females and males as we assumed this procedure to be too stressful for pregnant females. Fourteen anatomical landmarks were used along the wings of animals with the support of the software TpsDig v.1.18 [31]. Two types of anatomical landmarks were used as a way to sample homologous portions of the wing: maximum curvature points (anatomical landmark 10) and tissue joints (other anatomical landmarks - Figure 1b). These anatomical landmarks provide realistic representations of different wing regions and can be identified in any specimen.

During our analysis, we did not use any anatomical landmark that could limit propatagium or plagiopatagium (Figure 1a). This happened because of the consequent difficulty in real delimitation of these structures, because hairs (propatagium insertion onto the shoulder) or arbitrary position of legs (plagiopatagium insertion onto the calcaneus) can generate a high variation in the relative position of such structures. By this means we assumed a dashed straight line from landmark 2 (Figure 1b) towards the body of the animal to estimate the shape of the propatagium, and in the anatomical landmark 6 (Figure 1b) towards the body to estimate the plagiopatagium shape to establish comparisons. For this, we standardized the length of these dashed straight lines taking into account the relative position of the anatomical landmark 1 (Figure 1b). Even though we have not fully sampled the propatagium and plagiopatagium, we were able to verify if the shape of these sampled wing portions differed between males and females by analyzing the relative position of the digit V and the forearm, which retrains the propatagium and plagiopatagium (Figure 1).

To test the amount of error variance that could possibly be related to the standardization method used to extend the bat wings before obtaining the images, we checked the repeatability of all anatomical landmarks in 28 individuals (13 males and 15 females) recaptured which had their wings photographed in other occasions during the study. For this, we used the intraclass correlation coefficient from an analysis of variance on the x and y coordinates of each anatomical landmark, considering the image derived from the first capture as ‘sample 1′ and the image derived from the recapture as ‘sample 2′. From this analysis we were able to verify the error in locating the anatomical landmark position and the differences between individuals. Once all anatomical landmarks presented excellent repeatabilities [32] across samples, ranging from of 0.91 to 0.99, we assumed that the method adopted to extend the wings was standardized throughout the study.

The wing shape variables were obtained from the superimposition of anatomical landmarks (procrustes algorithm) using the software TpsRelW v.1.18 [33]. This method involves the centralization and minimization of distances between anatomical landmarks and the standardization of anatomical landmarks configuration from the Centroid Size (CS) [34], [35]. The CS is a multivariate measurement of size of the structure analyzed. This value is obtained by the square root of the sum of the square distance of each anatomical landmark to the mass center of each configuration (centroid) [35]. This isometric estimator of variation of wing sizes was also obtained from the software TpsRelw 1.18.

### Classical Morphometrics and Wing Area Measurements

For classical morphometric analysis, we followed the method adopted in ref. [9] (related to wing measurements) that also verified sexual dimorphisms in some bat species, including *S. lilium*. To this end, based on the same images used in geometric morphometrics analysis (Figure 1), we measured: **1)** Length of digit I – linear distance from anatomical landmark 3 to distal most point of first digit including claw; **2)** Length of digit III - linear distance from anatomical landmark 3 to anatomical landmark 14; **3)** Length of digit IV - linear distance from anatomical landmark 3 to anatomical landmark 9; **4)** Length of digit V - linear distance from anatomical landmark 3 to anatomical landmark 6. **5)** Length of forearm – linear distance from anatomical landmark 1 to anatomical landmark 3; **6)** Relative wing length – Linear distance from anatomical landmark 1 to anatomical landmark 14. This last measurement was also taken as another measurement of wing size (see *Statistical Analysis*).

In a similar way, areas of different portions of bat wings were measured from the same images used for geometric morphometric analysis. To this end, we measured the tissue area within polygons presented in Figure 1b and, therefore, we were able to confirm if differences found in wing shape were also related to different portions of wing area of males and females. All measurements were calculated using the Analyzing Digital Images Software v.2008 [36].

### Statistical Analyses

The use of body weight is of great importance to our study, once this variable supports our main hypothesis of sexual dimorphism in *S. lilium*. Thus, we checked if the weight (dependent variable) of pregnant females, non-pregnant females and males (categorical variables) is different using ANOVA coupled with Student’s post-hoc *t*-test.

In order to check if males and females (categorical variables) differ according to wing shape variables (partial warps and uniform components) obtained with the software TpsRelW v.1.18 (dependent variables) we performed a Hotelling’s T^2^ test. Subsequently, to maximize separation into groups of males and females, we performed a Linear Discriminant Analysis (LDA) using shape variables obtained with the software TpsRelW v.1.18. This analysis was conducted with the addition of CS of wings to assess whether this variable contributes to the correct reclassification of groups.

The analysis of correct classifications was checked by the stepwise classification through klaR package [37] of the software R 2.13.1 [38], to verify which variables contributed more for correct classifications through addition (step forward) and removal (step backward) of wing shape variables and CS. Similarly, we used the leave-one-out cross validation method to allow an estimate of the percentage of correct classifications that are not biased [39] using the ipred package [40]. This method consists in using the entire data set, with exception of a specimen, to calculate the discriminant function. Thus, the specimen not used in the analysis is classified. This procedure is repeated with all animals to compute the probability of individuals classified belonging to the correct group.

To graphically access differences in wing shape between sexes associated with the Canonical Variate (CV) derived from LDA, shape variables were regressed onto the CV scores. For visualization of major modifications in the bat wings, corresponding shapes for the extreme of the CV axis were generated using the software TpsRegr v.1.38 [41].

Overall differences between sexes regarding length and area measurements were verified using a Hotelling’s T^2^ coupled with Student’s post-hoc *t*-test. For this analysis we used the measurements described in the *Classical morphometrics and wing area measurements* and body length as response variables. After this, we compared the total wing area between males and females (N = 72) using a *t*-test separately, as this variable presented high correlation with the plagiopatagium (r = 0.91; P<0.001), dactylopatagium medius (r = 0.91; P<0.001), and dactylopatagium major (r = 0.91; P<0.001). Furthermore, we used a *t*-test to check if the CS of bat wings differs between sexes.

To estimate the influence of size in wing shape, wing length measurements and wing area measurements of *S.lilum*, we firstly performed two LDA (one for wing length measurements and one for wing area measurements) using these variables to obtain a CV that summarizes information contained in the set of original variables. Subsequently, we performed a multiple regression using the generated scores derived from these LDA and the scores derived from LDA of wing shape as dependent variables, and the CS, the relative wing length and the body length as independent variables.

## Results

We were able to identify significant differences between the body mass of males (N = 30), pregnant females (N = 14) and non-pregnant females (N = 42) (F_2,83_ = 7.161; *P* = 0.001). According to Student’s post-hoc *t*-test (P<0.05), pregnant females (Mean ± Standard Deviation/Standard Error; 24.7 g ±4.4 g/1.18 g) have greater body weight than non-pregnant females (21.2 g ±3.2 g/0.49 g) (P = 0.001) and males (22.2 g ±1.8 g/0.33 g) (P = 0.001), while non-pregnant females do not differ from males (P = 0.132).

**Figure 2:**
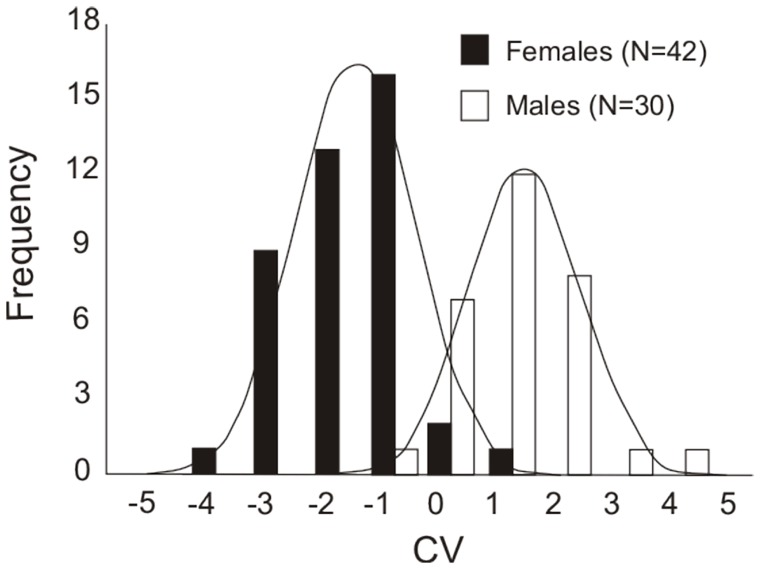
Histogram of scores generated by the Linear Discriminant Analysis (LDA) using wing shape variables of males and females of *Sturnira lilium*. The number in parentheses indicates the number of individuals of each sex analyzed.

The wing shape of 30 males and 42 females of *S. lilium* were analyzed. Our analyses of anatomical landmarks resulted in 24 wing shapes variables. The Hotelling’s T^2^ test with these variables revealed differences between males and females (T^2^_24,47_ = 4.098; P<0.0001). At performing LDA with wing shape variables and CS (Wilk's λ = 0.323; F_24,47_ = 4.103; P<0.0001), we obtained 80% of correct reclassifications (leave-one-out cross validation) of specimens, but the CS did not contribute to such classifications (stepwise classification −91% of correct reclassifications). The frequency of males and females according to scores generated in LDA can be checked in Figure 2.

**Figure 3:**
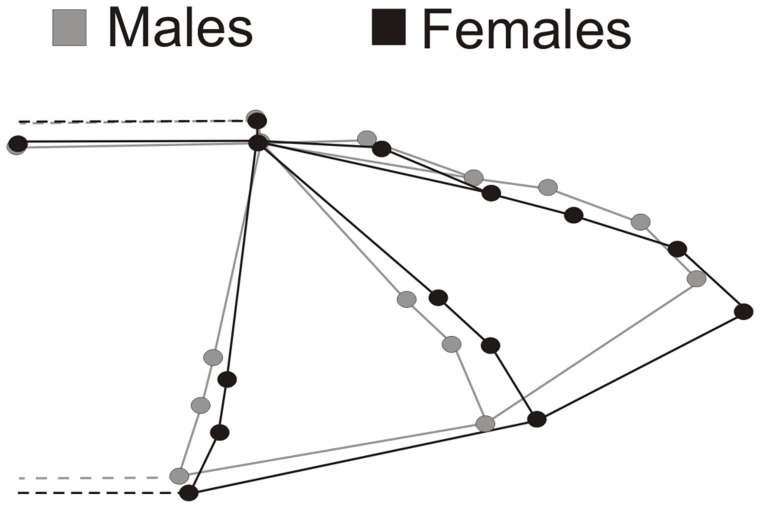
Canonical Variate (CV) analysis of wing shape variables (partial warps and uniform components) of males and females of *Sturnira lilium*. Major modifications in wing shape were obtained with the extreme of the CV from multivariate regression of shape variables onto CV scores.

Major wing modifications represented by the CV regressed onto shape variables (Figure 3) showed that while females have a displacement of the anatomical landmarks 7, 8 and 9 (Figure 1b) towards the dactylopatagium medius (Figure 1a), males have a displacement of these anatomical landmarks towards the plagiopatagium (Figure 1a). Additionally, we were able to observe a displacement of the anatomical landmarks 4, 5 and 6 towards the plagiopatagium for males, while females have these anatomical landmarks displaced towards the dactylopatagium medius. We observed a similar pattern in the dactylopatagium medius (Figure 1a). The relative position of anatomical landmarks 12, 13 and 14 (Figure 1b) revealed a wingtip more triangular for females and rounded for males (Figure 3). The relative position of the anatomical landmark 10 and 11, which represents the dactylopatagium minus (Figure 1b), also contributed to a less triangular shape in females and resulted in a wider shape when compared to males (Figure 3).

Overall differences between males and females of *S. lilium* were observed in wing length and area measurements as well as body length (T^2^_12,59_ = 2.160; P = 0.027). However, the pair-wise *t*-test comparisons revealed that the plagiopatatagium and the dactylopatagium minus, medius and major of females have larger areas compared to males, but wing length measurements, body length and propatagium area did not differ between sexes (Table 1). We also found differences in the total wing area between males (Mean ± Standard Deviation/Standard Error; 49.0±4.5/0.8) and females (53.7±4.7/0.7) (*t*_1_,_70_ = 3.203; P = 0.002), but CS differences between sexes were not significant (*t*_1,70_ = 0.349; P = 0.733).

**Table 1:** Results for pairwise comparisons of wing length measurements, body length and wing area measurements between 30 males (M) and 42 females (F) of *Sturnira lilium*, according to Student’s post-hoc *t*-test (Hotelling T^2^ test).

Measurement	Structure measured	Sex	Mean (SD/SE)	P
**Length (cm)**	Forearm	M	3.9±0.1/0.01	0.602
		F	3.9±0.1/0.01	
	Digit I	M	0.9±0.1/0.02	0.357
		F	0.9±0.1/0.02	
	Digit III	M	8.0±0.2/0.03	0.47
		F	8.0±0.2/0.03	
	Digit IV	M	6.3±0.2/0.02	0.435
		F	6.2±0.1/0.03	
	Digit V	M	5.9±0.2/0.04	0.711
		F	5.9±0.2/0.03	
	Relative wing length	M	11.7±0.4/0.10	0.151
		F	11.6±0.2/0.03	
	Body	M	5.8±4.4/0.80	0.859
		F	5.7±2.8/0.40	
**Area (cm^2^)**	Plagiopatagium	M	18.5±2.1/0.40	0.010
		F	19.9±2.1/0.30	
	Propatagium	M	2.4±0.4/0.10	0.162
		F	2.6±0.3/0.10	
	Dactylopatagium major	M	13.5±1.4/0.30	0.020
		F	14.4±1.2/0.20	
	Dactylopatagium medius	M	13.3±1.1/0.20	0.001
		F	14.5±1.1/0.20	
	Dactylopatagium minus	M	2.1±0.2/0.10	0.001
		F	1.1±0.3/0.10	

Wing length measurements were obtained following methodology adopted in ref. [9] (see *Classical morphometrics and wing area measurements* in *methods* for more details). Area measurements were calculated separately for different portions of the wing according to Figure 1. SD = Standard Deviation; SE = Standard Error; Adopted α = 0.05.

We found no allometric effects between the variables of size (CS, relative wing length and body length) and the CV derived from the wing shape, wing area measurements and wing length measurements (F_3,68_ = 1.044; R^2^ = 0.031; P = 0.290).

## Discussion

Even though we found differences in the wing shape of *Sturnira lilium* related to sex, our data indicated that there is no dimorphism related to animal size (i.e. body or wings). Our analyses also confirm that the CS of males and females’ wings do not differ statistically and did not contribute to the correct classification in the LDA. Therefore, our results suggest that the difference in the wing shape of males and females is not related to characteristics regarding the size of wings or body measurements, but it is related to a different distribution of area in distinct portions of the wings. This is supported by our results of wing areas (Table 1), which indicated larger areas in portions of the wing of females where we found major differences in shape (plagiopatagium and dactylopatagium major, medius and minus - Figure 3), while no differences in shape and area were found in the propatagium. Additionally, the lack of allometric effects also supports this idea, indicating important differences in the wing morphology regardless of the size of the body or wings.

Frugivorous bats, such as *S. lilium*, naturally exhibit a maneuverable flight as they normally inhabit complex environments with several obstacles [4]. Moreover, they usually carry fruits to perches before feeding themselves [42] and, therefore, wing loading for these bats should be smaller than open-area species that do not feed on fruits (i.e. insectivorous bats) [4]. The addition of an extra weight caused by the fetus or infant might compromise some aspects of the animal’s flight dynamics [20]. In our study, we found that pregnant females have a significant increase in body mass (about 16.5%) when compared to non-pregnant females. Taking into consideration that the fetus were still not fully developed, this increase of body mass in females should be grater at birth time. An increase in wing loading increases minimum flight speed, lowering the maneuverability [43] and, therefore, females would likely have a faster flight and greater energy expenditure as a way to maintain flight. Nevertheless, our results suggest different flight patterns between males and females. The increase in the area of specific portions of the wing found in females, responsible for generating and maintaining the thrust range for flight, should increase the efficiency in the use of generated forces and reduce energy expenditure. However, further studies considering experimental measurements of flight performance could confirm these conclusions.

Our limitation in analyzing the entire plagiopatagium could result in omission of features related to wing loading and flight performance in both sexes. However, our results revealed wider shapes (Figure 3) and larger areas (Table 1) in the portion analyzed of plagiopatagium and in the dactylopatagium major (Figure 1a) in females compared to males. The additional load allocated in the proximal portion of the wing, caused by fetus mass, may have great influence on the efficiency and dynamics of flight in females [20]. A larger area allocated in the plagiopatagium and in the dactylopatagium major could generate a smaller wing loading in these regions when females are pregnant or carrying newborns.

Additionally, triangular wingtips in females (Figure 3) are probably related to the need to increase the flight speed due to the additional weight caused by fetal mass or newborn carried during breastfeeding. Wings with pointed tips generate less friction with the air and, consequently, increase flight speed [44] without needing to increase wing flapping rate. Even though this type of flight can compromise the maneuverability of the animal, the larger area found in this portion of the wing (Table 1 and Figure 3) can probably minimize this flight aspect and at the same time contribute for the decreasing of wing loading.

The triangular wingtip in insects, birds and bats represents an evolutionary outcome that increases energy efficiency during the migratory flight [2], [45]. Bowlin and Wikelski [46] observed a positive relation between the heartbeat rate of the migratory bird *Catharus ustulatus* in function of their rounded wingtips. Therefore, it is possible that females have an increased efficiency in energy expenditure due to their more pointed wingtips in opposition to the more rounded wingtips of males. Additionally, the displacement of anatomical landmarks 10 and 11 (Figure 1b) towards the dactylopatagium medius (Figure 1a) in females can also contribute to a less rounded shape in this wing portion, thus, reducing air friction, which results in a faster flight with an increased efficiency in energy expenditure. Furthermore, the shape of the dactylopatagium minus clearly resulted in a larger area allocated in this portion of the wing (Figure 3), which was confirmed by our area measurements (Table 1).

An alternative hypothesis for a different wing shape between sexes would state that females have different wing shapes to accommodate foraging in different habitats as a resource partitioning pressure. Our results lead to an interpretation of a faster and yet maneuverable flight for females compared to males, which could indicate adaptations for pursuing insects between the canopies [5]. However, previous studies revealed a primarily or entirely frugivorous diet for this bat [46]–[48] with an increase of consumption of insects only when fruits are less abundant [47], and no evidence of intersexual differences in the diet of *Sturnira lilium* [46], [47]. In addition to this, once our results indicate that the shape is the main determinant for differences in wing between sexes and that there is no dimorphism related to size in *S. lilium*, the proposed hypotheses on secondary sexual dimorphism [24], at least for this species, are not supported.

This is the first study that uses superimposition of anatomical landmarks in a species of Phyllostomidae. Our results on the wing shape of *S. lilium* are particularly interesting because, up until now, no secondary sexual dimorphism was confirmed for this species. According to ref. [9], this species has no dimorphism related to any of the 12 cranial and 10 external characteristics of individuals sampled in the Caatinga (xeromorphic biome) and in the Cerrado at the Northeast region of Brazil. This is probably because of the methods used, which were insufficient to detect more subtle morphological variations. The lack of differences between sexes in our results of wing length measurements (Table 1), which were also used in ref. [9], confirms this. Therefore, classical approaches to check morphometric differences between males and females might mask important differences in wings and that have significant impact in interpretations related to lifestyle and ecology of these animals.

In accordance to ref. [25], the method for analyzing the variation in distinct points of the animal’s wing from partial and relative warps seems to have a greater power to detect differences among groups, as well as a greater graphic capability to detect and interpret such differences. But more interestingly is that our study provided evidence of morphological differentiation where traditional morphometrics have failed (in this study and ref. [9]), indicating possible missing features in other bat species. This method can be particularly useful for evaluation and re-evaluation of ecological inferences or flight dynamics in species of bats that have never been studied and those that have already been studied.

Thus, in conclusion, the enlargement or redistribution of wing areas, in both dactylopatagium and plagiopatagium (at least when taking into account the portion analyzed of the plagiopatagium), suggests greater thrust range to maintain flight, leading to a better use of this generated force in maintenance of flight by means of wing shape modifications. Therefore, differences in the distribution of specific areas in the wing seem to reflect an adjustment to pressures related to a higher energy requirement during gestation of females. Moreover, these morphologic features may be related to the transport of newborns during lactation which may also increase wing loading, decrease maneuverability and foraging efficiency [5], [23], and increase energy expenditure [49]. Further studies taking into consideration experimental measurements and analysis of flight performance of *S. lilium* could confirm our conclusions.
