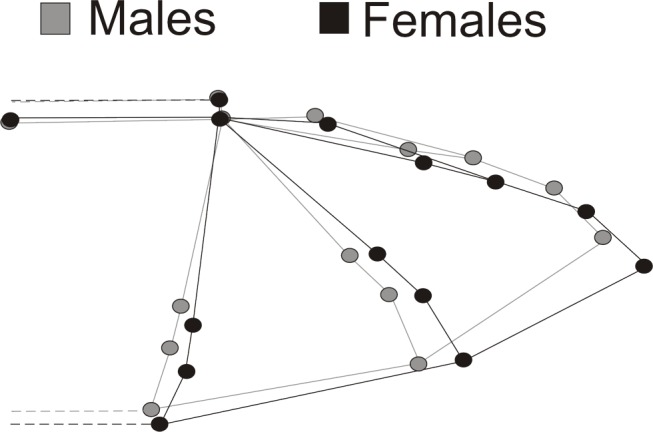# Correction: Sexual Dimorphism in *Sturnira lilium* (Chiroptera, Phyllostomidae): Can Pregnancy and Pup Carrying Be Responsible for Differences in Wing Shape?

**DOI:** 10.1371/annotation/ad74801b-b9f8-482f-914a-16e14b481913

**Published:** 2013-06-18

**Authors:** Nícholas F. de Camargo, Hernani F. M. de Oliveira

There are errors in Figures 1 and 3. Please see the corrected figures at the following links:

Figure 1 - 

**Figure:**
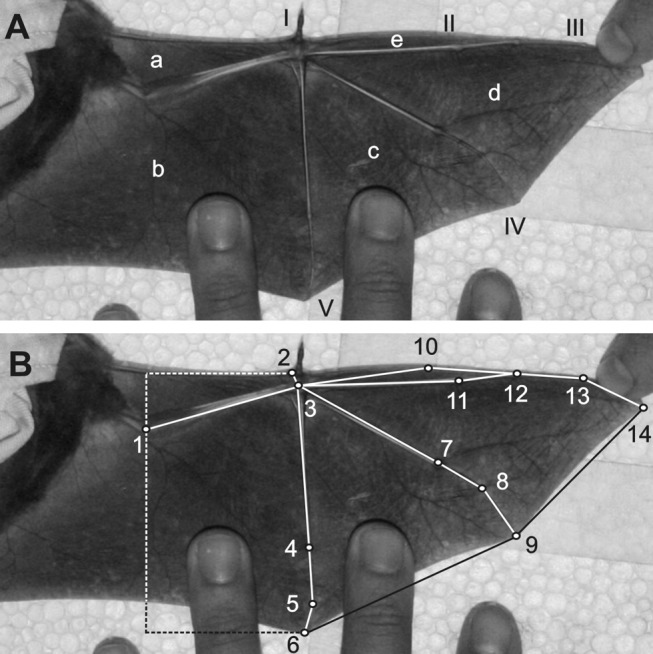


Figure 3 - 

**Figure:**