# Correction: Sexual Dimorphism in *Sturnira lilium* (Chiroptera, Phyllostomidae): Can Pregnancy and Pup Carrying Be Responsible for Differences in Wing Shape?

**DOI:** 10.1371/annotation/be3f20a6-177e-4e75-8217-cabc16b6d49e

**Published:** 2013-06-19

**Authors:** Nícholas F. de Camargo, Hernani F. M. de Oliveira

There is a statement missing from the Acknowledgments. The missing statement is:

We want to thank Ludmilla M. S. Aguiar for all the support during this study.

The Funding Statement incorrectly states that the authors received no financial support for their work. The correct funding statement is the following:

This work was supported by Foundation of the Federal District (FAPDF). The funders had no role in study design, data collection and analysis, decision to publish, or preparation of the manuscript. 

